# Cathepsin S-mediated autophagic flux in tumor-associated macrophages accelerate tumor development by promoting M2 polarization

**DOI:** 10.1186/1476-4598-13-43

**Published:** 2014-03-02

**Authors:** Min Yang, Jingwei Liu, Jianghua Shao, Yanwen Qin, Qunsheng Ji, Xiaolin Zhang, Jie Du

**Affiliations:** 1 Beijing Anzhen Hospital, Capital Medical University, Beijing, China; 2 The Key Laboratory of Remodeling-Related Cardiovascular Diseases, Capital Medical University, Ministry of Education, Beijing Institute of Heart Lung and Blood Vessel Diseases, Beijing 100029, China; 3 The Second Affiliated Hospital to Nanchang University, Jiangxi 330006, China; 4 Innovation Center China, AstraZeneca, Shanghai 201203, China

**Keywords:** Tumor-associated macrophages, Tumor microenvironment, Cathepsin, Autophagy

## Abstract

**Background:**

Tumor-associated macrophages (TAMs) are the major component of tumor-infiltrating leukocytes. TAMs are heterogeneous, with distinct phenotypes influenced by the microenvironment surrounding tumor tissues, but relatively little is known about the key molecular in these cells that contribute to malignant phenotypes. Autophagic activity is a critical factor in tumor development that contributes to enhancing cellular fitness and survival in the hostile tumor microenvironment. However, the molecular basis and relations between autophagy and TAMs polarization remain unclear.

**Methods:**

Cathepsin S (Cat S) expression was analyzed in human colon carcinoma and normal colon tissues. *In vivo* effects were evaluated using PancO2 subcutaneous tumor model and SL4 hepatic metastasis model. Immunofluorescence staining, flow cytometry and real-time PCR were done to examine TAMs polarization. Western blotting assay, transmission electron microscopy, mCherry-GFP-LC3 transfection and DQ-BSA degradation assays were carried out to determine its role in regulating autophagy.

**Results:**

In the present study, we showed that the enhanced expression of Cat S correlated with the severity of histologic grade as well as clinical stage, metastasis, and recurrence, which are known indicators of a relatively poor prognosis of human colon carcinoma. Cat S knockout led to decreased tumor growth and metastasis. Moreover, Cat S knockout inhibited M2 macrophage polarization during tumor development. We further demonstrated that Cat S was required for not only autophagic flux but also the fusion processes of autophagosomes and lysosomes in TAMs. Importantly, we found that Cat S contributed to tumor development by regulating the M2 phenotype of TAMs through the activation of autophagy.

**Conclusions:**

These results indicated that Cat S-mediated autophagic flux is an important mechanism for inducing M2-type polarization of TAMs, which leads to tumor development. These data provide strong evidence for a tumor-promoting role of autophagy in TAMs and suggest Cat S could be a potential target for cancer therapy.

## Background

Smouldering inflammation is a component of the tumor microenvironment, has recently been considered a hallmark of cancer with an important role in tumor initiation and progression [1,2]. In tumor microenvironment, innate immune cells are highly represented, and among the most abundant of these are macrophages. Although the original hypotheses proposed that macrophages are involved in antitumor immunity, there is substantial clinical and experimental evidence that in the majority of cases these tumor-associated macrophages (TAMs) enhance tumor progression to malignancy [3]. TAMs are heterogeneous in response to environmental signals and generally exhibit similarities with prototypic polarized M2 macrophages, contributing to tumor growth, invasion and angiogenesis [3-5]. Human clinical studies have shown a role of TAMs as tumor promoters based on the association of increased density of TAMs with tumor vascularization, metastasis, and poor prognosis [6-10], which have served as a paradigm for cancer-related inflammation. The link between TAMs and tumor development is well established. However, the mechanisms of TAMs moving toward a tumor-promoting phenotype are not fully understood.

Autophagy is an evolutionarily conserved, catabolic process that involves the entrapment of cytoplasmic components within characteristic vesicles for their delivery to and degradation within lysosomes [11-13]. The role of autophagy extends beyond the general homeostatic removal, degradation, and recycling of damaged proteins and organelles to many specific physiological and pathological processes such as development, immunity, energy homeostasis, cell death, tumorigenesis, among others [14-16]. Autophagy is a multifaceted process, and alterations in autophagic signaling pathways are frequently found in cancer [17-19]. While the involvement of autophagy in tumor development is widely accepted, it remains incompletely understood.

It is generally suggested that endolysosomal proteases play important roles in the degradation and regulation events of autophagic processes [11]. Cathepsins are cysteine lysosomal proteases that are essential for the turnover of intracellular and extracellular proteins internalized by endocytosis; cathepsins are now recognized that cysteine proteases play pivotal roles in cancer progression [20]. Of the cysteine cathepsins, B, L and S have been implicated most in serving as prognostic markers in cancer associated with poor outcome [21-25]. Cathepsin S (Cat S), unlike the ubiquitous cathepsin B (Cat B) and cathepsin L (Cat L), exhibits a restricted tissue expression. It is found predominantly in lymphatic tissue, macrophages, and other antigen-presenting cells [26]. There is an increasing body of data highlighting the upregulation of Cat S in a spectrum of tumors [23-25,27], where levels of the protease increase with the grade and aggressiveness of disease [28,29]. Studies previously demonstrated that macrophage-secreted Cat S plays a key role in tumor progression [30]. Recently, we further elucidated that Cat S deficiency results in abnormal accumulation of autophagosomes in macrophages and enhances Angiotensin II–induced cardiac inflammation [31]. However, whether Cat S-mediated autophagic flux promotes tumor development via the induction of TAMs polarization remains unclear. Thus, understanding the complex catabolic reactions that occur in the endolysosomal compartment is crucial for elucidating the mechanisms underlying TAMs-mediated tumor-promoting effects.

In this study, we identified that the expression of Cat S by TAMs is critical for promoting tumor growth and metastasis *in vivo*. We showed that Cat S deletion significantly blocked polarizing macrophages to the M2 phenotype within the tumor microenvironment. Moreover, we observed that Cat S deficiency led to the accumulation of autophagosomes and attenuation of autophagic flux in macrophages within the tumor microenvironment. Finally, we demonstrated that Cat S-mediated autophagic flux was pivotal to maintain polarization of the M2 TAMs phenotype.

## Results

### Cat S expression is elevated in human cancer tissue

**Figure 1:**
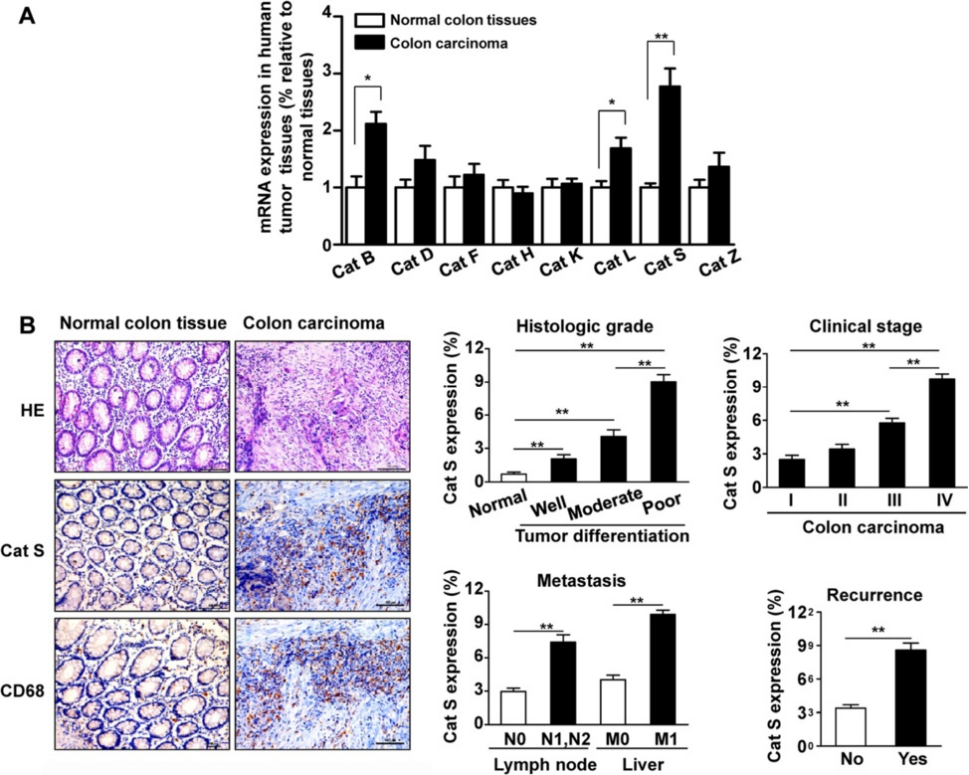
**Expression of Cat S in tumor tissues. (A)** Quantitative real-time PCR analysis of the mRNA level of Cathepsin family members in human colon carcinoma and adjacent normal colon tissues (samples from 15 human adenocarcinoma tissues and the healthy adjacent tissue). GAPDH was a normalization control. **, *P*<0.01 vs adjacent normal colon tissues. **(B)** HE staining and immunohistochemical analysis of Cat S or macrophage marker CD68 expression in normal colon tissues and human colon carcinoma. Histopathological differentiation grade indicates well-, moderately- and poorly-differentiated. T indicates tumor invasion depth. N indicates lymph node metastasis. M indicates distant metastasis. (×200 magnification and Scale bars = 100 μm). Quantitative analysis of Cat S expression in tumor sections at different histologic grade (well, moderate and poor), different tumor stages (I–IV;) and with or without recurrence 3 years after operation (n = 5-10 samples per group, with 10 fields per samples). **, *P*<0.01.

To determine whether cathepsin levels are increased in human colon carcinoma, real-time quantitative PCR was performed to measure the expression of cathepsin family members in human colon carcinoma and adjacent normal colon tissues. As shown in Figure 1A, the mRNA levels of Cat B, Cat L and Cat S were significantly elevated in cancer specimens compared with the matched normal tissues. Especially, analysis by quantitative real-time PCR revealed Cat S was prominently expressed and highly enriched in colon carcinoma specimens compared to healthy tissues, while the mRNA expression of Cat D, Cat F, Cat H, Cat K or Cat Z was not significant difference between colon carcinoma and normal colon tissues (Figure 1A). Immunohistochemical staining demonstrated that the expression of Cat S in adjacent normal colon tissues was low, whereas Cat S was observed in inflammatory-like cells located in carcinoma tissues (Figure 1B, *left panels*). Cat S expression was significantly elevated in poorly differentiated colon carcinoma, which was markedly higher than that of moderately differentiated or well differentiated colon carcinoma (Figure 1B, *left panels*). Moreover, we observed that Cat S was expressed in a high percentage of stage III and IV tumors (Figure 1B, *right panels*). Analysis of Cat S expression in primary tumors and metastases formation revealed that patients who had advanced clinical stage disease, metastasis, or recurrence within 3 years showed increased Cat S expression in colon cancer tissue (Figure 1B, *right panels*). Thus, Cat S expression correlates with human colon cancer aggressiveness.

Furthermore, advanced colon carcinoma tissues were markedly and extensively infiltrated by CD68-positive macrophages, particularly along the tumor cell-invasive front, while few macrophages were observed in normal colon tissues (Figure 1B, *left panels*). Cat S was expressed mainly in stromal macrophages as verified by staining with CD68, demonstrating that Cat S localizes to macrophages in stromal compartments of solid tumors. Cat S-positive cells within the area colocalized with CD68-positive macrophages, indicating a high concordance of CD68 and Cat S expression in human primary colon carcinoma during malignant progression.

### Cat S deficiency inhibits tumor growth and metastasis

**Figure 2:**
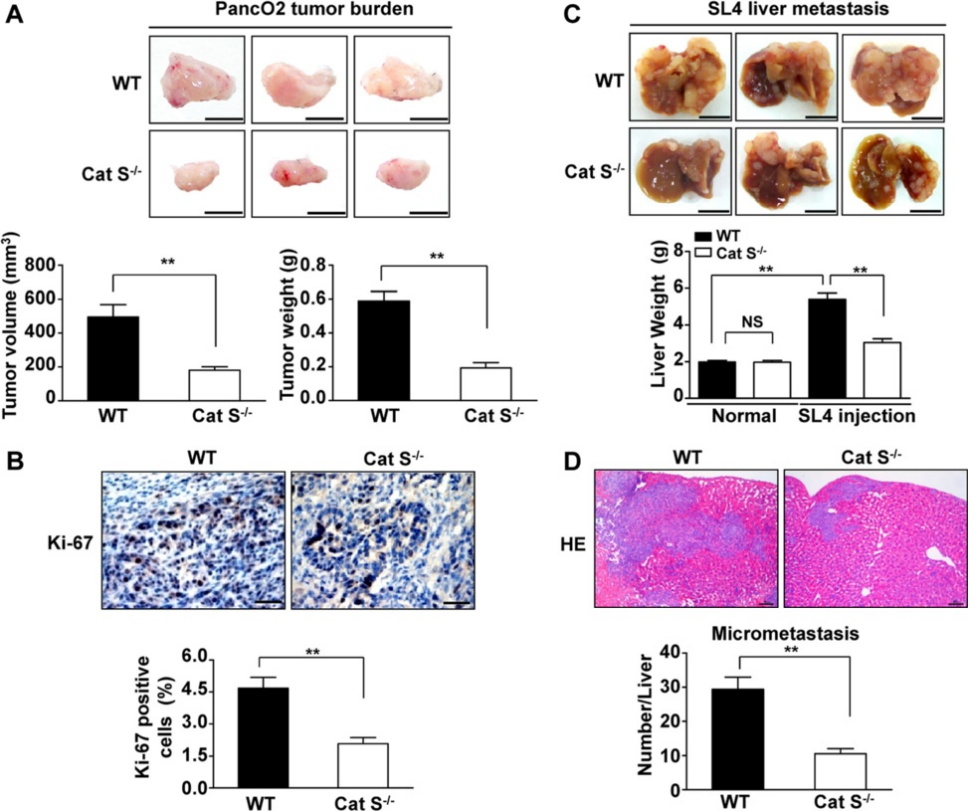
**Cat S deficiency inhibits tumor growth and metastasis. (A)** Representative images of tumors of WT and Cat S^-/-^ mice 28 days after subcutaneously inoculation of PancO2 cells. A suspension of 1 × 10^6^ PancO2 cells was injected subcutaneously into the flank of anaesthetized WT and Cat S^-/-^ mice. Mice were killed 28 days after transplant, and tumors were weighted after dissection. Bar = 1 mm. Data are mean ± SEM for n = 10 mice **, *P*<0.01. **(B)** Immunohistochemical analysis of Ki-67 expression in PancO2 tumor burden into WT and Cat S^-/-^ mice. (×400 magnification and Scale bars = 50 μm). Quantitative analysis of Ki-67 positive cells in tumor sections. Data are mean ± SEM for n = 10 mice with 10 fields per animal. **, *P*<0.01 vs WT mice. **(C)** Gross examination of development of liver metastasized tumor of colon cancer after intrasplenic injection of SL4 cells in WT and Cat S^-/-^ mice. SL4 cells (1 × 10^6^) were injected into the spleen of WT and Cat S^-/-^ mice. Mice were sacrificed at 14 days after tumor injection to determine the incidence of liver metastasis and tumor weight. The normal livers from WT or Cat S^-/-^ mice as control groups. Data are mean ± SEM for n = 10 mice **, *P*<0.01. **(D)** The analysis of micrometastasis was performed on paraffin-embedded sections with HE staining in metastasized foci after intrasplenic injection of SL4 cells in WT and Cat S^-/-^ mice. Number of micrometastatic lesions per one representative cross section of the livers from each mouse. (×100 magnification and Scale bars = 100 μm). Data are mean ± SEM for n = 10 mice with 10 fields per animal. **, *P*<0.01.

In order to investigate the roles of Cat S in the tumor microenvironment, we analyzed the effects of Cat S knockout on tumor growth and metastasis using subcutaneous and metastatic tumor models. In PancO_2_ subcutaneous tumor model, we found that Cat S knockout substantially suppressed tumor volume and reduced tumor weight (Figure 2A). The average tumor volume in the WT mice increased to 495.3 ± 73.17 mm^3^ for 28 days after PancO_2_ cells subcutaneous injection, whereas that in Cat S^-/-^ mice decreased to 181.0 ± 20.06 mm^3^, a significant inhibition of tumor growth (*P*<0.01). Moreover, immunohistochemistry showed that Ki-67 expression in PancO2 subcutaneous tumors was significantly lower in Cat S^-/-^ mice than in WT mice (Figure 2B). Previous studies have shown that SL4 cells injected intrasplenically formed tumors in the livers of mice [32], thus the SL4 cell line is a useful tool to study the process of tumor metastasis. To investigate the role of Cat S in liver metastasis of colon carcinoma cells, SL4 cells were injected into the spleens of WT and Cat S^-/-^ mice.

As shown in Figure 2C, there was no difference in normal liver weight between WT and Cat S^-/-^ mice. Fourteen days after SL4 injection, gross inspection demonstrated a marked increase of liver weight in WT mice due to multiple hepatic tumor nodules compared with weight of normal liver, whereas livers from Cat S^-/-^ mice showed fewer tumor foci and decreased tumor-occupied weight relative to that in WT mice (Figure 2C). To further investigate whether Cat S knockout attenuates tumor metastasis, the metastatic potential was quantified by scoring micrometastasis to livers in H&E-stained sections. We observed the number of metastatic foci was markedly decreased in Cat S^-/-^ mice versus WT mice on HE staining (Figure 2D), indicating a strong suppression in the metastatic potential of tumor cells. To investigate whether suppressing tumor development in Cat S^-/-^ mice could be caused by inhibition of angiogenesis, the sections were immunostained using the CD31 antibody (a marker of endothelial cells) to assess angiogenesis in tumor tissues. As shown in Additional file 1: Figure S1A, immunostaining for endothelial cells (CD31) demonstrated reduced microvessel density in PancO2 subcutaneous tumor from Cat S^-/-^ mice compared with that of in WT mice. Consistently, the density of CD31-positive microvessels from metastasized foci after intrasplenic injection of SL4 cells was significantly smaller in Cat S^-/-^ mice than those in WT mice (Additional file 1: Figure S1B). Therefore, the absence of stroma-derived Cat S inhibited tumor growth, metastasis and angiogenesis, supporting a tumor-promoting role of infiltrating leukocytes expressing Cat S.

### Cat S deficiency inhibits transition toward the M2 phenotype in TAMs

**Figure 3:**
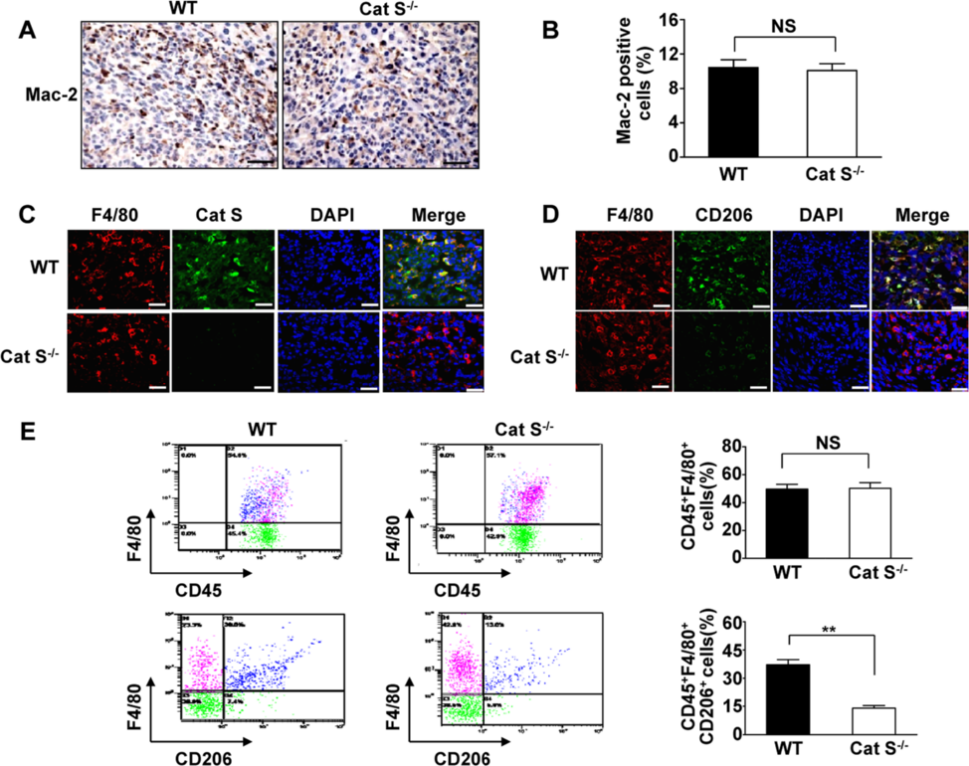
**Cat S deficiency reduces M2 macrophage infiltration in tumor development. (A)** Immunohistochemical analysis of macrophage infiltration in metastasized foci after intrasplenic injection of SL4 cells in WT and Cat S^-/-^ mice. Macrophage infiltration detected by anti-Mac-2 immunostaining (×400 magnification and Scale bars = 50 μm). **(B)** Quantitative analysis of Mac-2 positive cells in metastasized foci sections. Data are mean ± SEM for n = 8 mice with 10 fields per animal. NS indicates not significant. **(C)** Double-color immunofluorescence analysis of macrophage and Cat S expression in metastasized foci from WT and Cat S^-/-^ mice. The sections were immunostained with anti-F4/80 and anti-Cat S antibodies. (×400 magnification and Scale bars = 50 μm). **(D)** Double-color immunofluorescence analysis of M2 macrophage expression in metastatic foci from WT and Cat S^-/-^ mice. The sections were immunostained using the combination of anti-F4/80 and anti-CD206 antibodies. (×400 magnification and Scale bars = 50 μm). **(E)** Leukocytes were gated with CD45 fluorescence, macrophage M2 marker (CD45^+^F4/80^+^CD206^+^) were detected by flow from metastatic foci in the liver after intrasplenic injection of SL4 cells in WT and Cat S^-/-^ mice. Data are mean ± SEM for n = 8 mice per group. NS indicates not significant. **, *P*<0.01.

To further analyze the cellular origin of Cat S expression in tumor-infiltrating leukocytes, tumor sections were immunostained with the macrophage marker Mac-2 to assess macrophage infiltration into the tumor stroma. Immunohistochemical staining demonstrated that the expression of Mac-2 in hepatic metastatic tumors did not significantly differ between WT and Cat S^-/-^ mice (Figure 3A and B). Moreover, Cat S was highly expressed in macrophages, while Cat S^-/-^ macrophages showed no Cat S expression (Figure 3C). Furthermore, we did not observe a significant difference in F4/80-positive cells between WT and Cat S^-/-^ mice in hepatic metastatic tumors (Figure 3C).

TAMs are prominent components of leukocyte infiltrates and are frequently polarized to an M2 phenotype, which is characterized by tumor-promoting properties [4,6,33]. To further establish whether Cat S is necessary for M2 macrophage polarization in the tumor microenvironment, we assessed the cell surface expression of CD206, a marker of M2 macrophage. Immunofluorescence analysis revealed that double-positive F4/80 and CD206 viable macrophages were abundant in the tumors of WT mice but not in Cat S^-/-^ mice (Figure 3D). We further analyzed the macrophage phenotypes in tumors using flow cytometric analysis. As shown in Figure 3E, the population of macrophages (CD45^+^F4/80^+^ cells) in hepatic metastatic tumors of WT mice was predominantly CD206-positive, while the proportion of CD45^+^F4/80^+^CD206^+^ cells was significantly reduced in the tumors of Cat S^-/-^ mice (13.99 ± 1.45% versus 37.09 ± 2.81% for CD45^+^F4/80^+^CD206^+^ cells, *P*<0.01) (Figure 3E). These results suggest that Cat S may drive macrophages toward the M2 phenotype in the tumor microenvironment.

### Cat S deficiency induces autophagosome accumulation in TAMs

**Figure 4:**
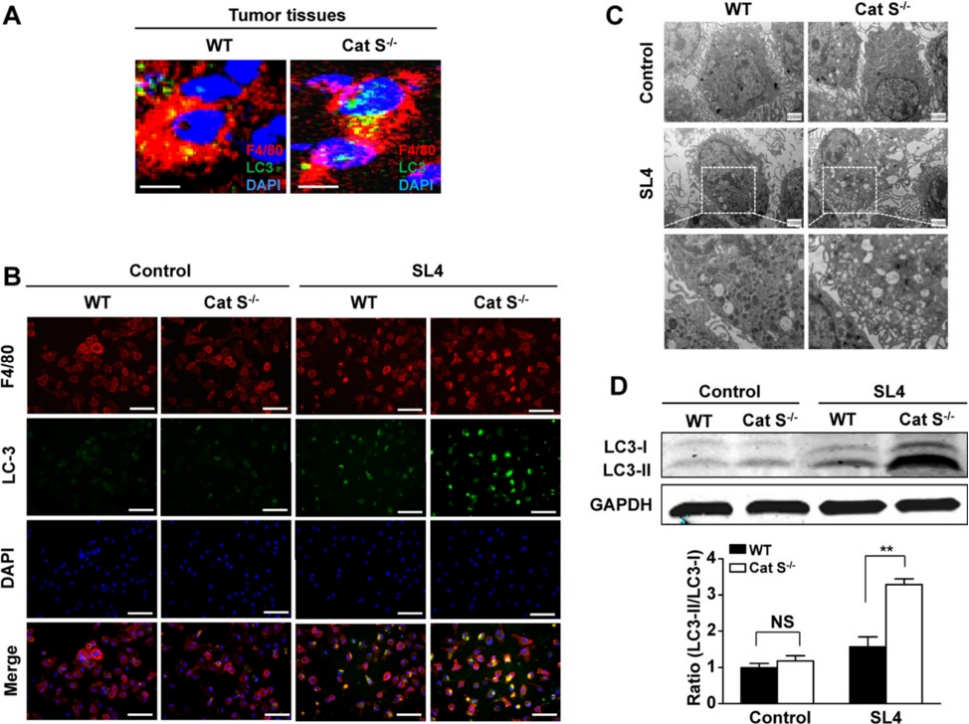
**Cat S deficiency increases accumulation of autophagosomes in macrophage in the tumor microenvironment. (A)** Double immunofluorescnce analysis of macrophage (anti-F4/80) and the autophagosome marker LC3 in metastatic foci from WT and Cat S^-/-^ mice. The sections were stained with anti-F4/80 (red) or anti- LC3 (green) antibody and DAPI (blue; to stain the nuclei). Scale bars = 10 μm. **(B)** Double immunofluorescnce analysis of macrophage (anti-F4/80) and the autophagosome marker LC3 in WT or Cat S^-/-^ macrophages cocultured with SL4 cells for 48 hrs and serum-starved for at least 12 hrs before co-culture. The sections were stained with anti-F4/80 (red) or anti- LC3 (green) antibody and DAPI (blue; to stain the nuclei). Scale bars = 40 μm. Three independent experiments were performed. **(C)** Ultrastructure was detected by transmission electron microscopy in WT or Cat S^-/-^ macrophages cocultured with SL4 cells for 48 hrs and serum-starved for at least 12 hrs before co-culture (×10000 magnification). Three independent experiments were performed. **(D)** Western blot analysis of LC3 protein expression in WT or Cat S^-/-^ macrophages cocultured with SL4 cells for 48 hrs and serum-starved for at least 12 hrs before co-culture. WT or Cat S^-/-^ macrophages cultured individually as control group. Quantitative analysis of LC3-II/LC3-I ratio in WT or Cat S^-/-^ macrophages cocultured with SL4 cells. Data are mean ± SEM of 3 independent experiments. **, *P*<0.01. NS indicates not significant.

Previous studies showed that autophagy is critical for malignant progression of tumors, serving to both reduce oxidative stress and provide key intermediates to sustain cell metabolism [34,35]. We recently reported that Cat S deficiency results in abnormal accumulation of autophagosomes in macrophages with angiotensin II treatment [31]. Therefore, we further analyzed whether the role of Cat S in the regulation of TAMs polarization was linked to autophagy. On immunofluorescence staining, we observed colocalization of the autophagosomal marker LC3 and macrophage marker F4/80 in the hepatic metastatic tumors of WT mice, and this effect was markedly increased in Cat S^-/-^ mice (Figure 4A).

To examine the expression of Cat S in macrophages, double-color immunofluorescence analysis was performed in bone marrow-derived macrophages. As shown in Additional file 2: Figure S2A, Cat S was expressed in F4/80-positive macrophages. Importantly, the level of Cat S expression in WT macrophages was markedly elevated after coculture with SL4 cells under serum-starved conditions. Western blotting demonstrated that SL4 cells significantly increased the level of Cat S protein in WT macrophages under serum-starved conditions (Additional file 2: Figure S2B). To further confirm the role of Cat S *in vitro*, confocal fluorescence microscopy showed that SL4 cells stimulated LC3 protein clustering in WT macrophages under serum-starved conditions, which was further enhanced in Cat S^-/-^ macrophages (Figure 4B).

To investigate whether autophagic structures were increased in Cat S^-/-^ macrophages in the tumor microenvironment, we detected autophagic vesicles by transmission electron microscopy (TEM). Autophagic vesicles are hallmarked by unique double membranes and by the presence of engulfed cytosolic content: features that allow them to be easily detected by TEM. Although WT or Cat S^-/-^ macrophages contained very few autophagic vesicles and did not differ in the formation of autophagosomes at baseline, SL4 cells induced cytosolic autophagic vacuole formation in serum-starved WT macrophages (Figure 4C). Especially, many large vesicles containing engulfed and digested membrane and organelle structures were observed in Cat S^-/-^ macrophages with SL4 cells coculture (Figure 4C).

The formation of phagophores and autophagosomes is associated with lipidation of LC3-I with phosphatidylethanolamine to produce the membrane-associated LC3-II protein [36]. We further measured autophagosome-associated LC3 (LC3 II) and free cytosolic LC3 (LC3-I) by western blotting. As shown in Figure 4D, in control, the LC3-II formation was in low level either WT or Cat S^-/-^ macrophages, no difference between the two groups. Consistent with autophagic vesicle accumulation, SL4 cells induced LC3-II formation in WT macrophages, which was even further enhanced in Cat S^-/-^ macrophages (Figure 4D). Our results suggest that Cat S deficiency may induce the accumulation of autophagosomes in macrophages with the tumor microenvironment.

### Cat S is required for autophagic flux of TAMs

The flux rate of autophagy can be controlled by several steps: 1) the enclosure of cytoplasmic components by phagophores to form autophagosomes; 2) the fusion of autophagosomes with lysosomes to form autophagolysosomes; 3) the dissolution of autophagolysosomes [11,15]. To investigate whether Cat S is involved in the initiation and progression of autophagy, autophagic flux was assessed using colocalization analysis of the tandem fluorescent mCherry-GFP-LC3 expression vector. In autophagosomes, both GFP and mCherry fluorescence is colocalized. In autophagolysosomes, the green fluorescence in the mCherry-GFP-LC3 fusion protein weakens and eventually disappears under acidic pH, making it a fluorescent sensor to simultaneously analyze autophagosomes and autophagolysosomes [37]. Progression from autophagosomes to autophagolysosomes, therefore, presents as less colocalization and stronger red fluorescence.

**Figure 5:**
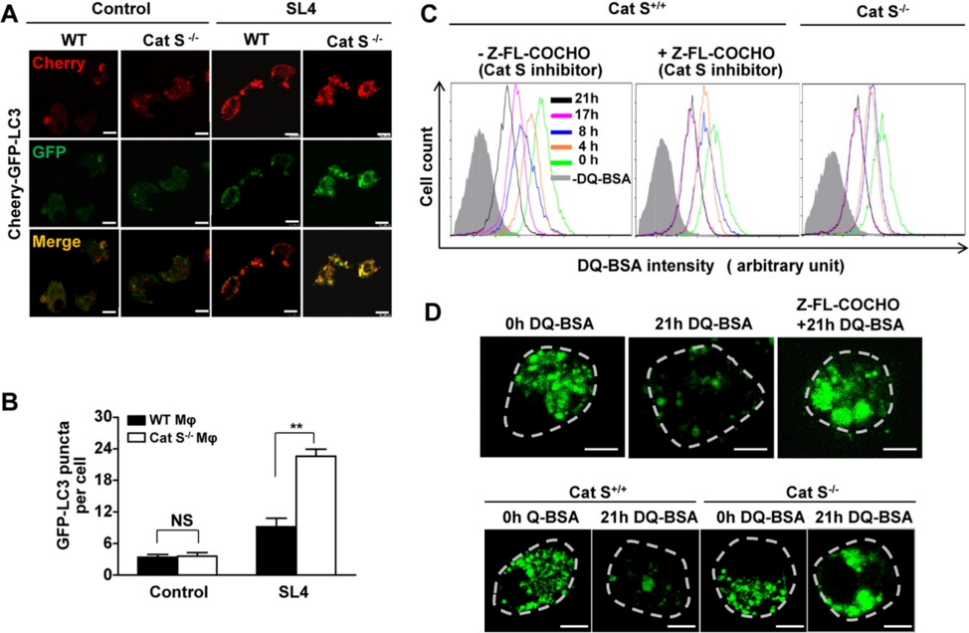
**Cat S inhibition causes defective autophagy flux in macrophage in the tumor microenvironment. (A)** WT or Cat S^-/-^ macrophages were transfected with Cherry-GFP-LC3, then cocultured with SL4 cells for 48 hrs and serum-starved for at least 12 hrs before co-culture. Confocal fluorescence was used to image autophagosomes (red and green foci) and autolysosomes (red-only foci). Scale bars = 10 μm. Three independent experiments were performed. **(B)** WT or Cat S^-/-^ macrophages expressing the GFP-LC3 reporter construct were seeded in plastic wells, then recognition and counting by fluorescence image analysis using the KineticScan HCS Reader. Data are mean ± SEM of 3 independent experiments. **, *P*<0.01. NS indicates not significant. **(C)** WT or Cat S^-/-^ macrophages were cocultured with SL4 cells and serum-starved for at least 12 hrs before co-culture, and WT macrophages in the presence or absence of Cat S inhibitor Z-FL-COCHO (10 μmol/L). WT or Cat S^-/-^ macrophages were loaded with 10 μg/mL DQ-Green BSA for 15 min, washed twice and incubated in media for the indicated times, then subjected to flow cytometry analysis. Background (gray peak) represents samples without the DQ-Green BSA loading. Three independent experiments were performed. **(D)** WT or Cat S^-/-^ macrophages were placed on coverslips as above in C, and incubated in media containing DQ-Green BSA (10 μg/mL) for 15 min. Cells were washed twice with PBS and incubated in media for the indicated times. Cells were fixed and the fluorescent degradation products of the DQ-Green BSA in lysosome were imaged using confocol images analysis. Scale bars = 5 μm. Dotted lines indicate the cell margin. Three independent experiments were performed.

In Cat S^-/-^ or WT macrophages, the lower basal level of autophagy presented as low-intensity fluorescence and was poorly localized (Figure 5A). We found that mCherry-GFP-LC3 expression was detected as predominantly red and yellow spots rarely observed in WT macrophages cocultured with SL4 cells, suggesting that the expression is mainly associated with autophagolysosomes (Figure 5A). In contrast, in Cat S^-/-^ macrophages cocultured with SL4 cells, yellow speckles with a higher level of GFP and mCherry fluorescence and their colocalization were significantly observed compared with those in WT macrophages (Figure 5A, *right panels*). The inhibition of fusion of autophagosomes with lysosomes resulted in the accumulation of mCherry-GFP-LC3 on autophagosomes; therefore, the number of GFP-LC3 puncta per cell was substantially higher in Cat S^-/-^ macrophages than in WT macrophages cocultured with SL4 cells, with no difference between the two cell types at baseline (Figure 5B). Our results indicated that Cat S deficiency inhibited autophagosome degradation to slow down the autophagy flux rate in TAMs.

An increase in autophagy should coincide with enhanced lysosomal activity and general proteolysis. We utilized the self-quenched reporter substrate, DQ^TM^ Green BSA (DQ-BSA) to measure of the cellular proteolytic/lysosomal activity. DQ-BSA-green is a bovine serum albumin (BSA) labeled with a self-quenching fluorescent dye. After DQ-BSA-green is delivered to lysosomes via endocytosis, it is hydrolyzed into single dye-labeled peptides by lysosomal proteases, thereby relieving self-quenching, and the green fluorescence can subsequently be monitored by flow cytometry [38]. Thus, DQ-BSA is useful for detecting intracellular proteolytic activity as a measure of a functional lysosome. To further examined whether Cat S deficiency could prevent autophagy flux, DQ Green BSA was pre-loaded and dequenched in macrophages, and the rate of disappearance of dequenched DQ-BSA was monitored. As shown in Figure 5C, *left panel,* fluorescent signals were decreased by approximately 90% over the time period of 21 hrs in WT macrophages cocultured with SL4 cells. However, in the presence of the Cat S inhibitor Z-FL-COCHO, fluorescence peaks shifted to the left was obviously inhibited, demonstrating that the flux of dequenched DQ-BSA was significantly delayed (Figure 5C, *middle panel*). Similarly, we observed the loss of the fluorescent signals was markedly blocked in Cat S^-/-^ macrophages compared with that in WT macrophages cocultured with SL4 cells, indicating that Cat S deficiency attenuated the DQ-BSA degradation rate (Figure 5C, *right panel*). Additionally, WT macrophages treated with the Cat S inhibitor or Cat S^-/-^ macrophages showed enlarged dequenched DQ-BSA-containing vesicles (Figure 5D, *top and bottom panels*). These results consistently suggested that Cat S is required for clearing dequenched DQ-BSA or flux of amphisomes.

### Cat S-mediated autophagy contributes to M2 phenotype polarization of TAMs

**Figure 6:**
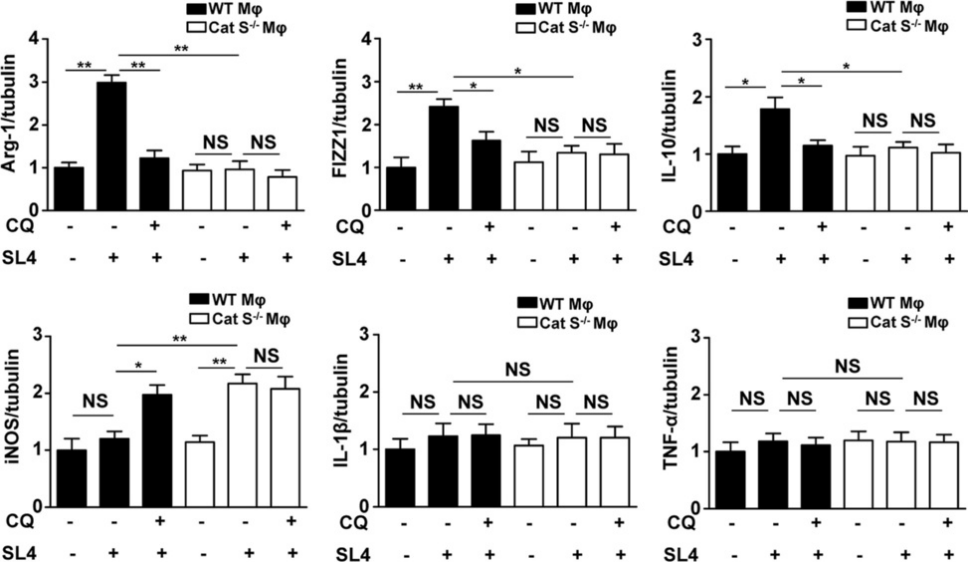
**Cat S is essential for macrophage autophagy contributing to M2-type polarization of macrophages.** WT or Cat S^-/-^ macrophages were pretreated with 50 μmol/L autophagy inhibitor (Chloroquine, CQ) or DMSO (vehicle) for 1 hr and then subjected to SL4 cells coculture, serum-starved for at least 12 hrs before co-culture. Quantitative real-time PCR analysis of the mRNA expression of M2 marker (Arg-1, FIZZ1, IL-10) or M1 marker (iNOS, IL-1β, TNF-α). Tubulin was a normalization control. Data are mean ± SEM of 3 independent experiments. *, *P*<0.05. **, *P*<0.01. NS indicates not significant. Mφ indicates macrophages.

To further confirm that Cat S-mediated autophagy plays an important role in macrophage polarization in the tumor microenvironment, we treated WT or Cat S^-/-^ macrophages with the autophagy inhibitor chloroquine (CQ) prior to coculture with SL4 cells, and assayed the expression of M2-type genes (arginase-1, Arg-1; found in inflammatory zone protein, FIZZ1 and interleukin-10, IL-10) and M1-type genes (inducible nitric oxide synthase, iNOS; interleukin-1β, IL-1β and tumor necrosis factor-α, TNF-α). As shown in Figure 6, coculture of SL4 cells markedly upregulated the mRNA levels of Arg-1, FIZZ1 and IL-10 (M2-specific genes) in WT macrophages, whereas CQ significantly inhibited the expression of Arg-1, FIZZ1 and IL-10 in WT macrophages cocultured with SL4 cells. Moreover, coculture of SL4 cell induced a significant increase in expression of Arg-1, FIZZ1 and IL-10 in WT macrophages compared to that of Cat S^-/-^ macrophages (Figure 6). Importantly, CQ treatment did not alter the expression of Arg-1, FIZZ1 or IL-10 in Cat S^-/-^ macrophages cocultured with SL4 cells (Figure 6, *top panels*). In addition, WT macrophages treated with CQ expressed higher levels of iNOS, displaying an M1-skewed profile when cocultured with SL4 cells (Figure 6). Cat S^-/-^ macrophages also showed a marked increase in the expression of iNOS compared with that in WT macrophages following coculture with SL4 cells, and this profile was not further altered in Cat S^-/-^ macrophages with CQ treatment (Figure 6, *bottom panels*). Taken together, Cat S-mediated autophagy promotes the polarization of TAMs toward an M2-like phenotype.

## Discussion

TAMs represent a dominant myeloid population in many solid tumors, and their accumulation correlates with poor prognosis [7,33]. TAMs-secreted cathepsin protease activity has been implicated in the pathogenesis of cancer [30], yet the mechanisms by it promotes tumor development are incompletely understood. In this study, we identified Cat S as the most abundantly expressed cysteine protease of the cathepsins family in tumor-infiltrating macrophages, and its level was associated with poor prognosis in human colon carcinoma. We showed that Cat S functions as a potentiator of tumor development through maintaining macrophage cellular homeostasis and mediating the switch to an M2 phenotype in TAMs. Moreover, we gained further insights into the potential mechanisms underlying Cat S as a critical mediator of autophagic flux, the cancer-promoting function of TAMs.

Clinical studies have shown that Cat S is expressed in 95% of cases of primary colorectal tumors and their related metastatic tissue, with significantly higher expression in tumors compared with matched normal colonic mucosa [39]. *In vitro* findings demonstrated that specific inhibition of Cat S by an antibody, Fsn0503, could attenuate colorectal cancer cell invasion [25]. Furthermore, these effects were confirmed in a murine model of sporadic pancreatic carcinogenesis (RIP1-Tag2), in which the genetic ablation of Cat S severally inhibited tumorigenesis through attenuation of tumor invasion and angiogenesis [23]. However, the mechanism by which stroma-derived Cat S promotes tumor development remains unclear. Therefore, in present investigation, we aimed to investigate the role and the mechanism of Cat S in regulating tumor microenvironment. In this study, we observed that colon carcinoma patients at more advanced clinical stages, those with lymph node or liver involvement, or those who have had recurrence within 3 years displayed markedly higher Cat S expression levels (Figure 1B). These results indicate a potential of Cat S as an independent or supplementary biomarker in the prediction of tumor prognosis. Furthermore, we found development of metastasized tumors in the liver and tumor growth of PancO_2_ cells were greatly inhibited in Cat S ^-/-^ mice compared to WT littermates (Figure 2), suggesting a critical role of TAMs Cat S expression in contributing to tumor development.

Besides of colon cancer tissues, previous studies have shown that significantly high levels of Cat S have been reported in a range of tumors including glioma [28], astrocytoma [24], lung cancer [40], prostate cancer [41], hepatocellular [27], and pancreatic carcinomas [42], revealing a possible role for this enzyme in tumour growth and progression. Cat S activity is also present in astrocytoma cells in vitro and the extracellular levels of activity were highest in cultures derived from grade IV tumors [24]. Furthermore, tumor cells such as breast cancer cells, pancreatic or colon cancer cells, express functional Cat S which has been proposed to involve in invasiveness of cancer cells [43,44].

In addition to the production of Cat S by the tumor cells, further analysis confirmed that TAMs produced the protease as previously shown in the mouse models for pancreatic cancer and breast cancer [23,30,45,46]. Several studies have shown that expression of Cat S by tumour-infiltrating macrophages, could be an important contributor during prostate cancer progression [41]. Recent studies have demonstrated that both tumor cells and tumor-associated macrophages can produce Cat S within the microenvironment to promote neovascularization and tumor growth [47]. In the present study, the data showed that Cat S deficiency impaired tumor angiogenesis in metastatic foci. Consistent with our results, Cat S null mice exhibited impaired endothelial microvessel development, suggesting a key role for this protease in angiogenesis [25]. Similarly, Cat S is markedly up-regulated by endothelial cells during tumour angiogenesis [27,42] and importantly, in a murine pancreatic islet carcinoma model (RIP1-Tag2), Cat S knockout mice have been shown to a significant reduction in tumour-associated angiogenic switching and neovascularisation [23]. Furthermore, the Cat S inhibitory antibody, Fsn0503, blocks extracellular proteolysis, inhibiting endothelial invasion and tube formation into developing tumors [48]. Taken together, reduced angiogenesis in Cat S deficient mice could also partially contribute to impaired tumor develpoment.

Lysosomal cysteine proteinases are known to mediate intracellular protein turnover, regulating the half-life of proteins critical for normal cell function. Cat S is restricted to lymphatic tissues and cells such as macrophages, indicating more specific roles in cell physiology [40,49,50]. Given that macrophages are major participants in host inflammatory responses, dysregulation of macrophage function may lead to diseases including autoimmune disease and cancer [3]. In the present study, we provide both *in vitro* and *in vivo* evidence that Cat S is required for M2 polarization and cancer-promoting functions of macrophages in the tumor microenvironment.

Autophagy is upregulated in response to stress, including growth factor and nutrient limitation, energy depletion, and hypoxia [12,51]. As a cell-refreshing and metabolism-supporting pathway, autophagy is required for the normal operation of cellular and organismal physiology. Therefore, through selective autophagy, the central metabolism may be supported by different substrates to restore metabolic and energy homeostasis, redox balance, and biomass production. Thus, autophagy can enable stress adaptation, maintenance of cellular fitness, and survival under different stress conditions [14]. In advanced cancers, autophagy can promote tumor progression by providing nutrients during starvation [14,16,52]. These findings suggest that autophagy inhibition, rather than stimulation, might be beneficial in the treatment of advanced cancer. Given the known importance of TAMs in cancer development and progression, the response of tumors to autophagy inhibition may also involve the immune system. One of the most important functions of autophagy is to maintain cellular energy under conditions of nutrient deprivation and other forms of stress [15]. The present study established a novel role of macrophage-secreted Cat S in the proper execution of autophagy. We showed that Cat S was required for efficient autophagic flux, based on the facts that Cat S-deficient macrophages showed more accumulation of autophagic vacuole-like structures and multivesicles than WT macrophages by serum starvation during SL4 cells coculture (Figure 4C). Moreover, significant higher levels of LC3-II were detected in Cat S-deficient macrophages than WT macrophages (Figure 4D). Autophagy is a vacuolar lysosomal degradation pathway for long-lived proteins and damaged organelles that are critical for maintaining cell function under stress conditions. During the late stage of autophagy, autophagosomes are presented to lysosomes to degrade damaged organelles (i.e., mitochondria) [12,53]. In the present study, Cat S deficiency increased autophagosome formation in macrophages in the tumor microenvironment (Figure 5A). Furthermore, we also showed that Cat S was required for the fusion processes of autophagosomes and lysosomes, as there were significantly defects in the dequenching process of DQ-BSA in Cat S inhibitor-treated macrophages and Cat S-deficient macrophages cocultured with SL4 cells (Figure 5C and D). These results suggest that the fusion of the autophagic vacuole with the lysosome and the subsequent degradation of its content by Cat S are crucial for proper execution of autophagy in TAMs.

Macrophages are highly heterogeneous cells that can be activated to a functional status between M1 and M2 phenotypes in response to environmental signals. In a simplified view, macrophages activated by LPS and IFN-γ are referred to as M1 macrophages, which are capable of killing pathogens and tumor cells [4,5]. Macrophages activated by IL-4, IL-13, and IL-10 are referred to as M2 macrophages, which can suppress inflammation, induce angiogenesis, promote tissue repair, and enhance tumor growth [4,5]. We demonstrated that the autophagy inhibitor CQ impairs the transition toward the M2 phenotype in macrophages within the tumor microenvironment. Importantly, lack of Cat S and CQ treatment did not produce additive suppression of M2 macrophage transition within the tumor microenvironment, further highlighting the biological necessity of Cat S-mediated autophagy in mediating M2-type polarization of TAMs.

Cat S, a cysteine protease of the papain family, is expressed in lysosomal/endosomal compartments of antigen-presenting cells, such as B cells, macrophages and dendritic cells (DCs) [54]. Inside the B cells and DCs, Cat S is the single enzyme during the assembly of the MHC class II-α and II-β chains with the antigenic peptide in the lysosomal/endosomal compartments [55]. This process contributes to antigen-induced adaptive immunity. Moreover, Cat S has been shown to dominate autoantigen processing in human thymic dendritic cells, which would be an important factor that influences selection of autoreactive T cells [56]. These findings suggest the role of Cat S in other immune cells could potentially also invovle in changes of our observed tumor development in this study.

## Conclusions

In summary, the activity of Cat S plays an important role in macrophage autophagy in the tumor microenvironment, leading to regulation of the M2 phenotype of TAMs. The results of the present study provide a strategy to selectively target inflammatory cells in combination with autophagy, an approach that could have significant therapeutic potential. Thus, controlling the activity of Cat S involved in autophagy, by developing drugs that target this process, can be considered as a future antitumor strategy.

## Materials and methods

### Antibodies and reagents

The antibodies for CD68, Ki-67, CD31, Mac-2, GAPDH, and IgG were from Santa Cruz Biotechnology (Santa Cruz, CA); the antibodies for Cat S, F4/80 and LC-3 was from Abcam (Cambridge, MA); and ChemMate TM EnVision System/DAB Detection Kits were from Dako (Glostrup, Denmark). Antibodies for PerCP/Cy5.5-conjugated CD45.2, phycoerythrin (PE)-conjugated F4/80, fluorescein isothiocyanate (FITC)-conjugated CD206 and isotype control were from Biolegend (San Diego, CA). Cat S inhibitor, Z-FL-COCHO was purchased from Calbiochem (San Diego, CA). Autophagy inhibitor, Chloroquine were purchased from Sigma (St. Louis, MO).

### Animals

The Cat S^-/-^ mouse strain was backcrossed onto the genetic background of C57BL/6 for more than 10 generations. Mice were 8–12 weeks old at the beginning of the experiments, matched for age and sex with wild-type (WT) mice, and kept under specific pathogen-free (SPF) conditions at the Beijing Anzhen Hospital affiliated to the Capital University of Medical Science, China. All animals received humane care in compliance with the Animal Management Rule of the Ministry of Health, People’s Republic of China (documentation no. 55, 2001) and the Care and Use of Laboratory Animals published by the US National Institutes of Health (NIH Publication No. 85–23, revised 1996) and approved by the Institutional Animal Care and Use and Committee of the Capital University of Medical Science (Beijing, China).

### Tumor model

PancO2 [57] and SL4 [32] cells are pancreatic and colon cancer cells, respectively, derived from C57BL/6 mice on the same background as the Cat S^-/-^ mice and WT control mice. PancO2 and SL4 cells were maintained in DMEM/F12 culture medium, supplemented with 10% FBS in a humidified 37°C incubator under 5% CO_2_.

For *in vivo* subcutaneous tumor model, PancO2 cells were harvested and single-cell suspensions of 1.0 × 10^6^ cells in 200 μl medium were injected subcutaneously into the right flank. Mice were sacrificed 28 days after subcutaneous injection. Tumor volume was measured with a caliper using the formula: V = π × [d^2^× D]/6, where d is the minor tumor axis and D is the major tumor axis. For *in vivo* hepatic metastasis model, after anaesthetizing mice, a transverse incision in the left flank was made, exposing the spleen, 1.0 × 10^6^ SL4 tumor cells in 100 μl DMEM/F12 medium were intrasplenically injected with use of a 26-gauge needle. 14 days after inoculation, mice were sacrificed, and the tissues were processed as described below. The spleen and liver were removed, wet spleen and liver weights were measured, and the incidence of liver metastasis was examined.

### Human colon carcinoma specimens

The specimens from 30 cases of human colon carcinoma tissue/adjacent normal colon tissues and the clinicopathologic data were obtained from the Second Affiliated Hospital to Nanchang University gastrointestinal tumor bank. The specimens were isolated at the time of surgery, formalin-fixed and paraffin-embedded, and stained with hematoxylin and eosin, then examined by 2 experienced pathologists. The clinicopathologic stage was determined according to the TNM classification system of the International Union against Cancer. RNA was extracted for fresh tissue specimens (from 15 colon carcinoma specimens and 15 adjacent normal colon tissues) with identifiable tumor in the tissue specimen. Human specimens use for research had been approved by the Second Affiliated Hospital to Nanchang University Research Ethics Committee.

### Histology and immunohistochemistry

Specimens were fixed for 24 hrs with 10% buffered formalin before embedding in paraffin. Serial sections of 5 μm thick were obtained for histologic analysis. Hematoxylin&eosin (HE) staining involved standard procedures.

For immunohistochemistry, sections were incubated with the primary antibodies for Cat S (1:200), CD68 (1:200), Ki-67 (1:200), CD31 (1:200), Mac-2 (1:200), then incubated with the Dako ChemMateTM EnVision System (Dako, Glostrup, Denmark) for 30 min. Staining was visualized with use of diaminobenzidine and counterstaining with hematoxylin. Negative controls were omission of the primary antibody, non-immune IgG or secondary antibody only; in all cases, negative controls showed insignificant staining. The expressions of Cat S, CD68, Ki-67, CD31, Mac-2 were calculated as proportion of positive area to total tissue area for all measurements of the section.

For double immunofluorescence, 7 μm frozen tissue sections were permeabilized and blocked with 0.1% Triton X-100, 0.2% bovine serum albumin, and 5% normal donkey serum in PBS, then incubated with the primary antibodies F4/80 (1:100), LC-3 (1:200) and Cat S (1:200) overnight at 4°C, then FITC or TRITC-conjugated secondary antibody (Jackson Immuno Research Laboratories, West Grove, PA, USA) at 4°C for 1 hr in the dark, and coverslipped with DAPI-containing mounting medium.

### RNA analysis

Total RNA were extracted by use of TRIZOL (Invitrogen, Carlsbad, CA, US). For reverse transcription, 1 μg of total RNA was used to generate first strand cDNA with Oligo-dT primer. Real-time PCR was performed using the SYBR Green Mix (Bio-Rad) on the CFX96 Real-time System (Bio-Rad). The Table 1 shows the primer used.

**Table 1:** Primers used for qRT-PCR

**Primer**	**Forward**	**Reverse**
Human cathepsin B	5′gtttgcattgctggtcagga3′	5′tggcaggacagtggaatgat3′
Human cathepsin D	5′gcgagtacatgatcccctgt3′	5′ctctggggacagcttgtagc3′
Human cathepsin F	5′ tggcaacaagatgaagcaag3′	5′ttttgtgacagcccccttac3′
Human cathepsin H	5′actggctgttgggtatggag3′	5′ aggccacacatgttctttcc3′
Human cathepsin K	5′ccgcagtaatgacacccttt3′	5′gcacccacagagctaaaagc3′
Human cathepsin L	5′acagtggaccaagtggaagg3′	5′cttctcccacactgctctcc3′
Human cathepsin S	5′tcatacgatctgggcatgaa3′	5′aggttctgggcactgagaga3′
Human cathepsin Z	5′aagggggtaatgacctgtcc3′	5′ttcattgcatgtcccacatt3′
Human GAPDH	5′acagtcagccgcatcttctt3′	5′acgaccaaatccgttgactc3′
Mouse Arg-1	5′aaagctggtctgctggaaaa3′	5′ acagaccgtgggttcttcac3′
Mouse FIZZ1	5′ttgcaactgcctgtgcttac3′	5′ctgggttctccacctcttca3′
Mouse IL-10	5′ccaagccttatcggaaatga3′	5′ttttcacaggggagaaatcg3′
Mouse iNOS	5′gggctgtcacggagatca3′	5′ccatgatggtcacattctgc3′
Mouse IL-1β	5′gcccatcctctgtgactcat3′	5′aggccacaggtattttgtcg3′
Mouse TNF-α	5′tcttctcattcctgcttgtgg3′	5′ggtctgggccatagaactga3′
Mouse Tubulin	5′tctaacccgttgctatcatgc3′	5′gccatgttccaggcagtag3′

GAPDH, glyceraldehyde 3-phosphate dehydrogenase; Arg-1, arginase-1; FIZZ1, found in inflammatory zone 1; IL-10, interleukin 10; iNOS, inducible NO synthase; IL-1β, interleukin 1β; TNF-α, tumor necrosis factor-alpha.

### Western blotting

Protein extracts were diluted with loading buffer and separated by electrophoresis on 8%-10% SDS-polyacrylamide gels before transfer to nitrocellulose membranes (Bio-Rad). The membranes were blocked in Odyssey blocking buffer (LI-COR Bioscience, Lincoln, NE) at room temperature for 1 hr, then incubated at 4°C overnight with primary antibodies: Cat S (1:1000), LC-3 (1:1000), GAPDH (1:3000). The membranes were washed 3 times in TBST and incubated with fluorescent secondary antibodies (Alexa Fluor 680 or IRDye 800, Rockland Immunochemicals, Gilbertsville, PA, US) for 1 hr at room temperature at 1:5000, blots were analyzed with the Odyssey infrared imaging system and Odyssey software.

### Flow Cytometry

The content of inflammatory cells was quantified by flow cytometry as described [58]. Briefly, tumor tissues were cut into multiple small cubes and digested in an enzyme mixture for 45 mins at 37°C. The cell suspension was centrifuged and pre-incubated with Fc-γ block antibody (anti-mouse CD16/32; Pharmingen, San Diego, CA, USA) to prevent nonspecific binding. Cell staining involved different combinations of fluorochrome-coupled antibodies to CD45, F4/80, CD206 for 30 mins at 4°C in the dark. Fluorescence data were collected by use of an EPICS XL flow cytometer (Beckman Coulter) and analyzed by use of Cellquest (Beckman). Fluorescence minus one (FMO) controls were included to determine the level of nonspecific staining and auto-fluorescence associated with subsets of cells in each fluorescence channel.

### Co-cultures of bone marrow-derived macrophages (BMDMs) and tumor cells

Co-culture systems were established by using transwell inserts (0.4 mm pore, polycarbonate membrane; Costar, Cambridge, USA) and transferred to 6-well culture plates. Bone marrow-derived macrophages (BMDMs) were isolated from tibias and femurs of 8-week-old wild-type C57BL/6 or Cat S^-/-^ mice as previously described [58]. SL4 cell suspensions (1 ml, 1 × 10^6^ cells) were loaded in the upper inserts, and WT or Cat S^-/-^ BMDMs suspensions (3 ml, 3 × 10^6^ cells) were put into the lower compartment of the culture well for 48 hrs and serum-starved for at least 12 hrs before co-culture. Serum-free DMEM/F12 without SL4 cell inserts was used as a control in the lower compartment of well. BMDMs were preincubated for 1 hr with 10 μmol/L Cat S inhibitor Z-FL-COCHO or 50 μmol/L autophagy inhibitor Chloroquine, immediately after SL4 cells plating and controls received equivalent dilution with DMSO vehicle alone.

### Transmission electron microscopy

For transmission electron microscopy (TEM), cells were fixed in 2% glutaraldehyde in 0.1 M sodium cacodylate buffer (pH 7.0) for 2 hrs, post-fixed in 2% osmium tetroxide for 2 hrs and then rinsed with 0.1 M cacodylate buffer. Cells were enrobed in 5% Noble Agar and washed with distilled water 5 times, further fixing with 2% uranyl acetate for 2 hrs, followed by dehydration in 50% (15 min), 70% (16 h), 85% (15 min), 95% (15 min), and 2 changes of 100% ethanol each 15 min. They were then cleared by 2 changes of propylene oxide, each 15 min, and infiltrated with epon resin:propylene oxide (1:1) for 3 hrs, epon resin:propylene oxide (3:1) for 16 hrs, and 2 changes with pure epon resin for total 6 hrs. Thin sections were mounted on grids and examined under the electron microscope (Philips EM410).

### Autophagy flux assays

#### mCherry-GFP-LC3 transfection

Cat S^-/-^ or WT BMDMs were transfected with mCherry-GFP-LC3 reporter construct by using Lipofectamine 2000 (Invitrogen, Carlsbad, CA) as previously described in our lab [31]. After transfection, Cat S^-/-^ or WT BMDMs cocultured with SL4 cells without serum for 48 hrs. Cells were fixed with 4% paraformaldehyde and microphotographs of mCherry-GFP-LC3 fluorescence were obtained with the confocal laser-scanning microscope (TCS 4D; Leica, Heidelberg, Germany). Treatment-induced changes in GFP intensity in mCherry-positive puncta were calculated to depict autophagic flux.

#### High-throughput image analysis

Cat S^-/-^ or WT BMDMs expressing GFP-LC3 were seeded in 96-well plates, then cocultured with SL4 cells in 100 μl of medium/well without serum for 48 hrs. Images data were collected with an ArrayScan HCS 4.0 Reader with a 20× objective (Cellomics) for hoechst-labeled nuclei and GFP-tagged LC3. The detection of punctated staining of GFP-LC3 from the diffuse staining indicated the formation of autophagosomes. Images of 1,000 cells for each well were analyzed to obtain the average cell number per field, fluorescence spot number, area, and intensity per cell.

#### DQ-BSA degradation assays

To quantify protein degradation induced by stimuli, autophagy flux was analyzed by flow cytometry and confocal microscopy using the self-quenched substrate DQ-Green BSA (Molecular Probes, Eugene, OR) as described in previous studies [59]. Briefly, WT or Cat S^-/-^ BMDMs were cocultured with SL4 cells for 48 hrs and serum-starved for at least 12 hrs before co-culture. WT BMDMs were in the presence or absence of Cat S inhibitor Z-FL-COCHO (10 μmol/L) cocultured with SL4 cells. WT or Cat S^-/-^ BMDMs into the lower compartment were loaded with 10 μg/ml DQ-Green BSA for 15 min at 37°C. The cells were washed twice with PBS to remove excess label, then harvested at indicated time points. Cells were harvested, and Green-fluorescent of DQ-BSA was analyzed by flow cytometry using a FACSCalibur flow cytometer (BD Biosciences) and CellQuest (BD Biosciences) and FlowJo (Treestar) software. For confocal images analysis, cells were placed on coverslips after treatment as above and fixed with 4% formaldehyde. The fluorescent degradation products of DQ-BSA in lysosomes were imaged using a confocal laser-scanning microscope (TCS 4D; Leica, Heidelberg, Germany).

### Statistics

Data were analyzed with GraphPad Prism v5.00 for Windows. Results are expressed as mean ± SEM. Differences were analyzed by *t* test or ANOVA, and results were considered significant at a *P*<0.05.

## Abbreviations

Cat S: Cathepsin S; Cat B: Cathepsin B; Cat D: Cathepsin D; Cat F: Cathepsin F; Cat H: Cathepsin H; Cat K: Cathepsin K; Cat L: Cathepsin L; Cat Z: Cathepsin Z; TAMs: Tumor-associated macrophages; Arg-1: Arginase-1; FIZZ1: Found in inflammatory zone protein; IL-10: Interleukin-10; iNOS: Inducible nitric oxide synthase; IL-1β: Interleukin-1β; TNF-α: Tumor necrosis factor α; CQ: Chloroquine.

## Competing interests

The authors declare that there are no conflicts of interest.

## Authors’ contributions

MY and JL equally participated in the design and execution of the overall study. JS carried out the pathologic analysis. YQ provided Cat S knockout mice and performed mice genotyping. QJ, XZ participated in its coordination and provided technical support. JD was involved in the conception and design of the study as well as in revising the manuscript. All authors read and approved the final manuscript.

## Supplementary Material

Additional file 1: Figure S1 Cat S deficiency inhibits angiogenesis in tumor development. The area of vessels was evaluated by immunohistochemical analysis with anti-CD31 antibody in PancO2 subcutaneous tumors (A) and in metastasized foci after intrasplenic injection of SL4 cells (B). (×200 magnification and Scale bars = 100 μm). Quantitative analysis of CD31 expression in subcutaneous tumors and metastasized foci sections. Data are mean ± SEM for n = 8 mice with 10 fields per animal. **, *P*<0.01. Click here for file

Additional file 2: Figure S2 SL4 cells induce Cat S expression in macrophages. (A) BMDMs were cocultured with SL4 cells for 48 hrs and serum-starved for at least 12 hrs before co-culture. Double immunofluorescence analyses of Cat S expression in BMDMs with or without SL4 cells treatment. BMDMs were stained with anti-F4/80 (red) or anti-Cat S (green) antibody and DAPI (blue; to stain the nuclei). (×400 magnification and Scale bars = 50 μm). Three independent experiments were performed. (B) Western blot analysis of the protein levels of Cat S in BMDMs with or without SL4 cells treatment. GAPDH was used as a loading control. Quantitative analysis of Cat S/GAPDH ratio in BMDMs with or without SL4 cells treatment. Data are mean±SEM of 3 independent experiments. **, *P*<0.01. Click here for file
