# Supplementary material for: Cathepsin S-mediated autophagic flux in tumor-associated macrophages accelerate tumor development by promoting M2 polarization
Source: Mol Cancer. 2014 Mar 2;13:43. doi: 10.1186/1476-4598-13-43 (PMC4015740; doi:10.1186/1476-4598-13-43)
Supplement: Additional file 1: Figure S1 — Cat S deficiency inhibits angiogenesis in tumor development. The area of vessels was evaluated by immunohistochemical analysis with anti-CD31 antibody in PancO2 subcutaneous tumors (A) and in metastasized foci after intrasplenic injection of SL4 cells (B). (×200 magnification and Scale bars = 100 μm). Quantitative analysis of CD31 expression in subcutaneous tumors and metastasized foci sections. Data are mean ± SEM for n = 8 mice with 10 fields per animal. **, P<0.01. [file 1476-4598-13-43-S1.pdf]

Supplementary Figure 1

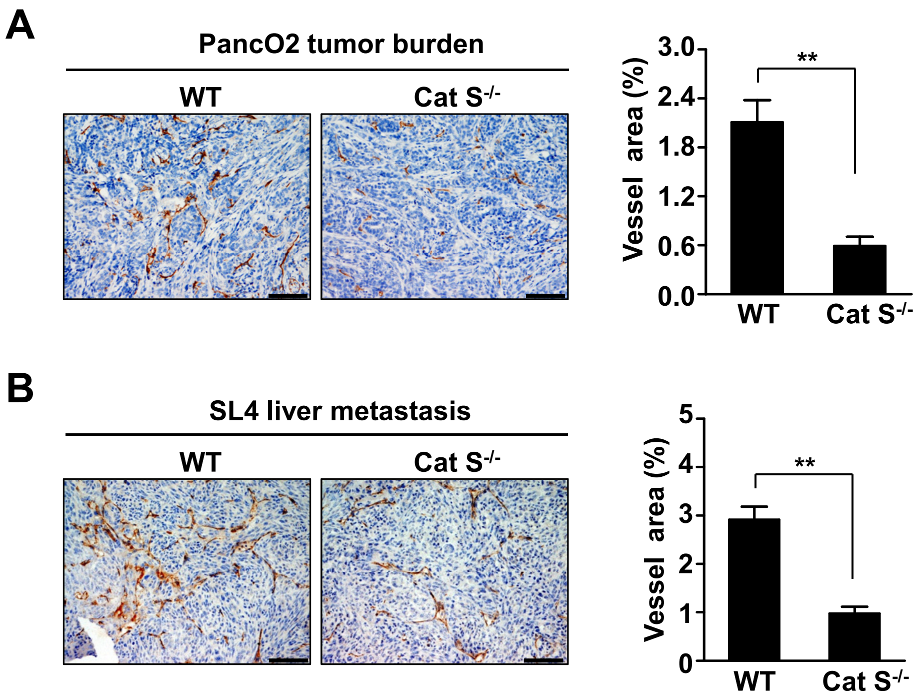

Supplementary Figure 1. Cat S deficiency inhibits angiogenesis in tumor development. The area of vessels was evaluated by immunohistochemical analysis with anti-CD31 antibody in PancO2 subcutaneous tumors (A) and in metastasized foci after intrasplenic injection of SL4 cells (B). (×200 magnification and Scale bars = 100 μm). Quantitative analysis of CD31 expression in subcutaneous tumors and metastasized foci sections. Data are mean ± SEM for n = 8 mice with 10 fields per animal. **, $P < 0.01$.
