# Supplementary material for: Cathepsin S-mediated autophagic flux in tumor-associated macrophages accelerate tumor development by promoting M2 polarization
Source: Mol Cancer. 2014 Mar 2;13:43. doi: 10.1186/1476-4598-13-43 (PMC4015740; doi:10.1186/1476-4598-13-43)
Supplement: Additional file 2: Figure S2 — SL4 cells induce Cat S expression in macrophages. (A) BMDMs were cocultured with SL4 cells for 48 hrs and serum-starved for at least 12 hrs before co-culture. Double immunofluorescence analyses of Cat S expression in BMDMs with or without SL4 cells treatment. BMDMs were stained with anti-F4/80 (red) or anti-Cat S (green) antibody and DAPI (blue; to stain the nuclei). (×400 magnification and Scale bars = 50 μm). Three independent experiments were performed. (B) Western blot analysis of the protein levels of Cat S in BMDMs with or without SL4 cells treatment. GAPDH was used as a loading control. Quantitative analysis of Cat S/GAPDH ratio in BMDMs with or without SL4 cells treatment. Data are mean±SEM of 3 independent experiments. **, P<0.01. [file 1476-4598-13-43-S2.pdf]

Supplementary Figure 2

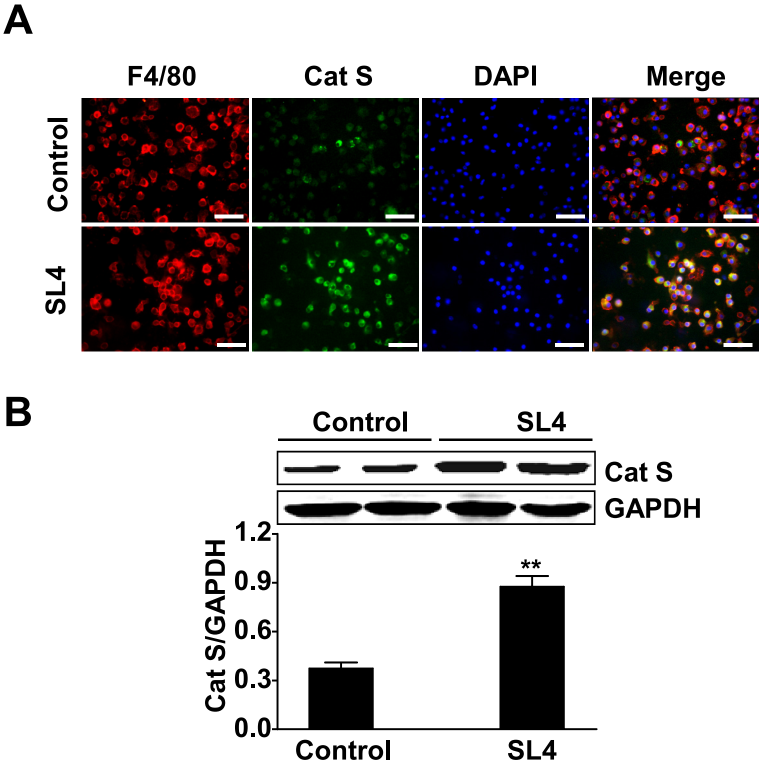

Supplementary Figure 2. SL4 cells induce Cat S expression in macrophages. (A) BMDMs were cocultured with SL4 cells for 48 hrs and serum-starved for at least 12 hrs before co-culture. Double immunofluorescence analyses of Cat S expression in BMDMs with or without SL4 cells treatment. BMDMs were stained with anti-F4/80 (red) or anti-Cat S (green) antibody and DAPI (blue; to stain the nuclei). (×400 magnification and Scale bars = 50 μm). Three independent experiments were performed. (B) Western blot analysis of the protein levels of Cat S in BMDMs with or without SL4 cells treatment. GAPDH was used as a loading control. Quantitative analysis of Cat S/GAPDH ratio in BMDMs with or without SL4 cells treatment. Data are mean±SEM of 3 independent experiments. **, $P<0.01$.
